# An anterior cruciate ligament injury does not affect the neuromuscular function of the non-injured leg except for dynamic balance and voluntary quadriceps activation

**DOI:** 10.1007/s00167-016-4335-3

**Published:** 2016-09-24

**Authors:** Tjerk Zult, Alli Gokeler, Jos J. A. M. van Raay, Reinoud W. Brouwer, Inge Zijdewind, Tibor Hortobágyi

**Affiliations:** 1 Center for Human Movement Sciences, University of Groningen, University Medical Center Groningen, A. Deusinglaan 1, 9700 AD Groningen, The Netherlands; 2 Department of Orthopedic Surgery, Martini Hospital, Groningen, The Netherlands; 3 Department of Neuroscience, University of Groningen, University Medical Center Groningen, Groningen, The Netherlands

**Keywords:** ACL deficient, Bilateral impairment, Force accuracy, Force variability, Maximal voluntary force, Postural balance, Proprioception, Twitch interpolation

## Abstract

**Purpose:**

The function of the anterior cruciate ligament (ACL) patients’ non-injured leg is relevant in light of the high incidence of secondary ACL injuries on the contralateral side. However, the non-injured leg’s function has only been examined for a selected number of neuromuscular outcomes and often without appropriate control groups. We measured a broad array of neuromuscular functions between legs of ACL patients and compared outcomes to age, sex, and physical activity matched controls.

**Methods:**

Thirty-two ACL-deficient patients (208 ± 145 days post-injury) and active and less-active controls (*N* = 20 each) participated in the study. We measured single- and multi-joint neuromuscular function in both legs in each group and expressed the overall neuromuscular function in each leg by calculating a mean z-score across all neuromuscular measures. A group by leg MANOVA and ANOVA were performed to examine group and leg differences for the selected outcomes.

**Results:**

After an ACL injury, duration (−4.3 h/week) and level (Tegner activity score of −3.9) of sports activity decreased and was comparable to less-active controls. ACL patients showed bilateral impairments in the star excursion balance test compared to both control groups (*P* ≤ 0.004) and for central activation ratio compared to active controls (*P* ≤ 0.002). There were between-leg differences within each group for maximal quadriceps and hamstring strength, voluntary quadriceps activation, star excursion balance test performance, and single-leg hop distance (all *P* < 0.05), but there were no significant differences in quadriceps force accuracy and variability, knee joint proprioception, and static balance. Overall neuromuscular function (mean z-score) did not differ between groups, but ACL patients’ non-injured leg displayed better neuromuscular function than the injured leg (*P* < 0.05).

**Conclusions:**

Except for poorer dynamic balance and reduced quadriceps activation, ACL patients had no bilateral neuromuscular deficits despite reductions in physical activity after injury. Therapists can use the non-injured leg as a reference to assess the injured leg’s function for tasks measured in the present study, excluding dynamic balance and quadriceps activation. Rehabilitation after an ACL injury should be mainly focused on the injured leg.

**Level of evidence:**

III.

## Introduction

An injury to the anterior cruciate ligament (ACL) compromises not only the injured but presumably also the non-injured limb’s function. Quadriceps weakness [31], impaired ability to fully activate the quadriceps muscle [43, 44], and difficulty in maintaining single-leg balance [34] can be present in both legs after an ACL injury up to even 2 years after reconstruction [16]. The function of the non-injured leg after the first ACL injury is clinically important because 8 % of the ACL reconstructed patients suffer a subsequent ACL injury to the non-injured leg, with an even higher risk for patients younger than 25 years (11 %) [48]. However, a comprehensive characterization of the non-injured leg’s neuromuscular function is lacking.

The non-injured leg is often used as a reference for the neuromuscular function of the injured leg, but it is likely that the neuromuscular deficit is underestimated if the status of the non-injured leg is also compromised [30, 35]. To determine the functional deficit in the non-injured leg after an ACL injury, it would be necessary to compare patient outcomes to an age, sex, and physical activity matched control group. In studies on ACL injuries, the physical activity level of control participants is often matched to the pre-injury activity level of ACL patients [31]. However, since the amount of physical activity decreases following the injury ACL patients’ leg function should be more appropriately compared against a less-active control group matched to the ACL patients’ post-injury activity level.

Quantifying the magnitude and nature of any neuromuscular deficit in the non-injured leg after an ACL injury is important because it can shed light on the neuromuscular scope of the injury, reduce the risk of a contralateral ACL injury if deficits are treated adequately, and inform therapists’ decision to treat the non-injured leg. Unfortunately, previous research has examined neuromuscular deficits in the non-injured leg for only a few neuromuscular measures (i.e. quadriceps strength, voluntary quadriceps activation, single-leg balance) [31, 34, 43, 44]. Therefore, the purpose of this study was to compare a broad array of neuromuscular measurements carried out on ACL patients’ injured and non-injured leg and compare these to the legs of active and less-active controls, while controlling for age, sex, and physical activity. The ACL patients’ non-injured leg was expected to demonstrate impaired neuromuscular function compared with active but not less-active controls. The largest decline in neuromuscular function was still expected to occur in ACL patients’ injured leg.

## Materials and methods

### Participants

Table 1 shows the group characteristics of the ACL-deficient patients awaiting surgery (16 men, 16 women) and healthy volunteers (20 men, 20 women). Patient inclusion criteria were: age 18–30 years, unilateral ACL tear with/without partial meniscal resection, and time between ACL injury and testing <2 year. Patient exclusion criteria were: previous ACL reconstruction, history of a lower limb injury that required surgery, pregnancy, current or prior neurological conditions. Controls were between age 18–30 years and had no history of orthopaedic, cardiovascular, neurological, and cognitive impairments. Controls were recruited via ads on social media, where we specifically asked for active and sedentary persons. After recruitment, controls were subdivided into an active and less-active group based on the physical activity level (i.e. hours spent on sport per week). The ten most active men and women were allocated to the active group, and the ten least active men and women were allocated to the less-active group. We have also quantified the level of physical activity through the Tegner activity score [42]. Leg dominance was determined using the Waterloo Footedness Questionnaire [12].

**Table 1:** Group characteristics (mean ± SD)

Group	*N*	Age (years)	Sex (male/female)	Leg dominance (right/left)	Mass (Kg)	Height (cm)	BMI (kg/m^2^)	Physical activity	Physical activity	Main sport
ACL patients	32	23 ± 4	16/16	29/3	77 ± 12	178 ± 9	24 ± 3	Pre-injury: hours/week: 6.9 ± 4.6 Tegner score: 8.1 ± 1.6	Post-injury: hours/week: 2.6 ± 2.6 Tegner score: 4.2 ± 1.4	Soccer (*N* = 20) Basketball (*N* = 3) Fitness (*N* = 3) Jogging (*N* = 0) Others (*N* = 5) None (*N* = 1)
Active controls	20	22 ± 2	10/10	19/1	73 ± 12	178 ± 11	23 ± 2	Hours/week: 6.6 ± 2.4 Tegner score: 7.7 ± 1.7	Hours/week: 6.6 ± 2.4* Tegner score: 7.7 ± 1.7*	Soccer (*N* = 10) Basketball (*N* = 0) Fitness (*N* = 2) Jogging (*N* = 2) Others (*N* = 6) None (*N* = 0)
Less-active controls	20	22 ± 1	10/10	16/4	73 ± 17	176 ± 10	23 ± 5	Hours/week: 2.5 ± 1.9* Tegner score: 5.4 ± 2.5*	Hours/week 2.5 ± 1.9 Tegner score: 5.4 ± 2.5	Soccer (*N* = 7) Basketball (*N* = 0) Fitness (*N* = 3) Jogging (*N* = 3) Others (*N* = 2) None (*N* = 5)

For the Tegner activity score, *α* was set at *P* < 0.017 (Bonferroni correction) to correct for multiple comparisons

* Different from all other groups (*P* < 0.05)

### General experimental protocol

As a warm-up, each participant started with 5 min of cycling on a bicycle ergometer. Next, maximal knee flexor and extensor strength, quadriceps force accuracy and variability, knee joint proprioception, voluntary quadriceps activation, static and dynamic balance, and single-leg hop distance were measured. Every participant performed every test with each leg randomized between legs.

### Maximal voluntary contraction (MVC)

Following strictly the manufacturer’s guidelines and our own previous protocols, we have measured isometric and dynamic (concentric and eccentric) quadriceps and hamstring MVCs on an isokinetic dynamometer (Biodex Medical Systems, Shirley, NY, USA) [7, 10, 11, 17, 27, 28]. Participants’ knee range of motion for the concentric and eccentric contractions was set between 0° (full knee extension) and 90° of knee flexion. After a thorough familiarization with the contraction conditions, participants performed three isometric MVCs at 65° of knee flexion [28], three eccentric MVCs at 60°/s, and six concentric MVCs each at 60, 120, and 180°/s. There was a 1-min pause between conditions. The order of quadriceps and hamstring contractions and the order of isometric and dynamic MVCs were alternated between participants. The peak torque value, normalized to body weight, was used in the statistical analysis.

### Voluntary quadriceps activation

Quadriceps activation was assessed with twitch interpolation and the central activation ratio (CAR) during isometric contractions [5, 31, 43, 44]. Participants were strapped to the seat of a custom-built dynamometer [46], with the hips and knees in 90° flexion and the arms folded in front of the chest. We have stimulated the quadriceps through two 10 × 14 cm aluminium foil electrodes, covered with water-soaked sponges (cathode: middle of rectus femoris, anode: distal 10 cm above patella), connected to a high-voltage stimulator (Digitimer DS7AH, Welwyn Garden City, UK) that discharged two pulses 10 ms apart (200-µs pulse, 100 Hz). We refer to the force evoked by a doublet as a twitch. The torque signal was amplified, sampled at 500 Hz (CED Power 1401 Plus; Cambridge Electronic Design, Cambridge, UK), visually inspected on a monitor, and recorded and offline-analysed by software (Spike 2, version 5.21). The protocol consisted of: 1. Three isometric quadriceps MVCs; 2. Maximal twitch torque determination during contractions at 10 % MVC (to remove slack); 3. Superimposed twitches at 30, 50, 75, and 100 % of MVC; 4. Two twitches at rest from which the higher of the two was classified as potentiated twitch.

**Fig. 1:**
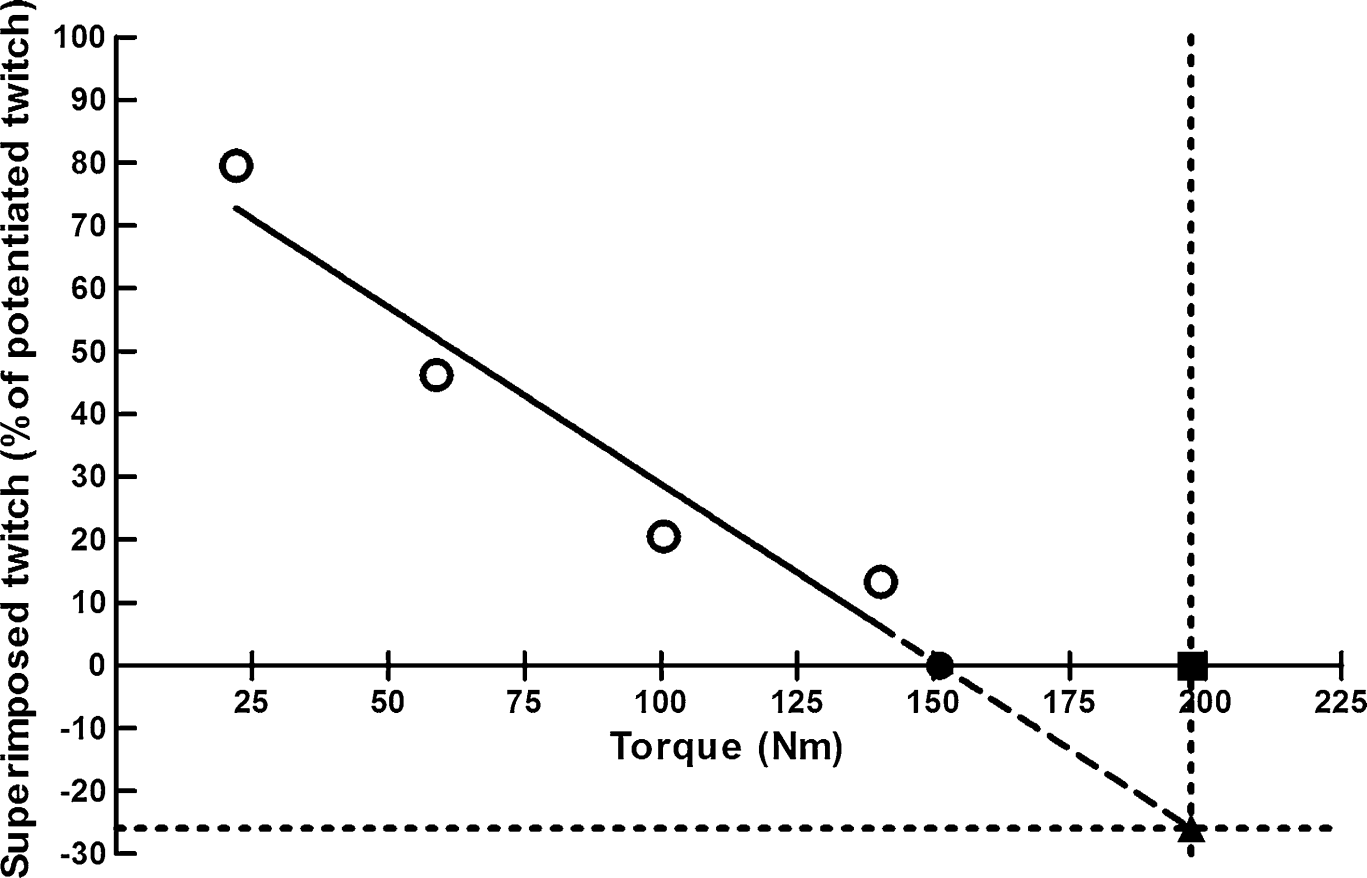
Voluntary quadriceps activation determined for a single subject using linear regression equation (*y* = −0.56*x* + 85.11; *R* = −0.96). The *open circles* represent the four data points used for calculating the linear regression equation. Intersection point with the *x*-axis is the estimated maximal torque (151.3 Nm, *filled circle*). Intersection point with the *y*-axis using the maximal quadriceps torque is the estimated quadriceps activation (−25.9 %, *filled triangle*). *Note* the estimated maximal torque underestimates the produced maximal torque (197.3 Nm, *filled square*)

At 10, 30, 50, and 75 % of MVC, we have computed a ratio as: (superimposed twitch/potentiated twitch) *100 %. The ratio for each contraction intensity was plotted against the respective force upon which the twitch was superimposed. A linear regression equation (*y* = *ax* + *b*) was then generated for each participant to determine the estimated maximal force and voluntary muscle activation (Fig. 1). The estimated maximal force was determined by calculating the intersection point with the *x*-axis, and voluntary activation was derived by determining the intersection point with the *y*-axis using the actual MVC torque [5]. The CAR was calculated as: MVC/(MVC + superimposed twitch) * 100 %.

### Force accuracy and variability

Participants have matched the produced torque as steadily and accurately as possible with the target torque displayed as a horizontal line on the monitor set to 20 % of MVC for the isometric trials and to 40 Nm for the dynamic trials [27, 28]. After familiarization, participants performed three isometric trials at 65° of knee flexion (5-s duration) and four concentric and eccentric trials at 20°/s between 90° and 10° of knee flexion. The order of dynamic and isometric contractions was rotated between participants. Force accuracy and variability were computed in the final 3-s portion of the data for isometric trials and the middle 2-s portion for dynamic trials. Force accuracy was the absolute difference between the produced torque and the target torque. Force variability was the coefficient of variation (i.e. SD of the produced force divided by the mean force). Force accuracy and force variability were calculated for each data point, and the average across the trials was used in the statistical analysis.

### Knee joint proprioception

Knee joint proprioception was measured, in a random order, at 15, 30, 45, and 60° of knee flexion using a joint repositioning task [27]. Knee joint proprioception was computed as the absolute difference between the actual leg position and the target position and was expressed in degrees.

### Static balance

Static balance was measured using the one-leg standing balance test, starting with eyes-open followed by eyes-closed condition [2]. The maximum score that participants could obtain was 60 s. The best score of the two trials was used in the statistical analysis.

### Dynamic balance

The star excursion balance test (SEBT) was used to assess dynamic balance [19]. The normalized scores from the eight directions were averaged to create a composite score used for the statistical analysis. After 5-min of rest, the measurement continued with the other leg as the stance leg.

### Single-leg hop test

Participants performed the single-leg hop test for distance, allowing the use of the arms to accelerate [9]. The hop distance was measured from the toe at push-off to the heel where the participant landed. The maximal hop distance was used in the analysis. All participants provided written informed consent to the experimental procedures, which were approved by the medical ethics committee of the University Medical Center Groningen (ID 2012.362) and in accordance with the Declaration of Helsinki.

### Statistical analyses

Data in the text and figures are presented as mean ± SD (SPSS version 22). Each variable was checked for normality. A one-way ANOVA was used to test for differences between groups in age, mass, height, BMI, and the amount of physical activity. Between-group differences in sex and Tegner activity score were tested using, respectively, a Chi-square and a Kruskal-Wallis test. A group (3) by leg (2) MANOVA was performed to test the between-leg differences in quadriceps MVCs (5 conditions), hamstring MVCs (5 conditions), voluntary quadriceps activation (5 conditions), force accuracy (3 conditions), force variability (3 conditions), proprioception (4 conditions), and static balance (2 conditions). Pillai’s Trace was used to determine between- and within-subject effects. A significant MANOVA was followed up by univariate ANOVAs. Dynamic balance and single-leg hop distance were analysed using a group by leg one-way ANOVA. In addition, we converted the outcome on every neuromuscular measure to a *z*-score. The *z*-scores were averaged per neuromuscular function (i.e. quadriceps MVCs, hamstring MVCs, voluntary quadriceps activation, force accuracy, force variability, proprioception, static balance, dynamic balance, and single-leg hop distance), and a mean *z*-score calculated across these nine functions was used to test the overall difference in neuromuscular function between legs. Significant *F* values from the ANOVA’s were subjected to a Tukey HSD post hoc pairwise comparison to determine the means that were different. The level of significance (*α*) was set at *P* < 0.05.

The sample size was based on a previous study reporting bilateral impairments in quadriceps strength and activation in ACL-deficient patients [31]. About 50 % more ACL-deficient patients were included compared to Lepley et al. [31], because our ACL patients would be less homogeneous with regard to the time since injury.

## Results

### Group characteristics

ACL-deficient patients were all recreational athletes, and 29 of 32 sustained a non-contact ACL injury, ruptured the ACL on the non-dominant side (*N* = 17), and reported relatively few knee complaints on a visual analogue scale (mean 28 ± 15, 0 no and 100 severe pain) [14]. The time between injury and testing was 208 ± 145 days (range 60–664 days) and between testing and surgery was 23 ± 17 days (range 2–62 days).

Table 1 shows the group characteristics. The groups did not differ in age, sex, mass, height, BMI, or leg dominance (all n.s.). Less-active controls had a lower Tegner score and a shorter duration of sport participation per week than ACL patients prior to injury and active controls (*P* < 0.01). In addition, these two variables were, respectively, 61 and 45 % lower for ACL patients after injury compared to active controls (*P* < 0.001).

### Single-joint neuromuscular function

Table 2 shows the static and dynamic quadriceps MVCs. The MANOVA showed a leg (*F*_5,65_ = 8.4, *P* < 0.001) and a group by leg interaction effect (*F*_10,132_ = 3.9, *P* < 0.001). Follow-up of univariate ANOVAs showed an interaction effect for all five MVC conditions (all *P* ≤ 0.018) caused by the greater between-leg differences in ACL patients than controls.

**Table 2:** Maximal voluntary contraction data of both legs of ACL-deficient patients and active and less-active controls (mean ± SD)

Variables	Group	Non-injured leg/dominant leg	Injured leg/non-dominant leg	Difference	
				Absolute	Percentage
Quadriceps (Nm/kg)					
Eccentric 60°/s	ACL patients	3.6 ± 0.8	3.1 ± 0.8	0.5^†^	13.9
	Active controls	4.0 ± 1.0	3.6 ± 0.8	0.4^†^	10.0
	Less-active controls	3.5 ± 0.9	3.5 ± 1.0	0.0	0.0
Isometric	ACL patients	3.5 ± 0.7	3.1 ± 0.8	0.4^†^	11.4
	Active controls	3.7 ± 0.6	3.6 ± 0.7	0.1	2.7
	Less-active controls	3.4 ± 0.7	3.2 ± 0.8	0.2^†^	5.9
Concentric 60°/s	ACL patients	2.5 ± 0.6	2.2 ± 0.6	0.3^†^	12.0
	Active controls	2.6 ± 0.6	2.6 ± 0.5	0	0.0
	Less-active controls	2.5 ± 0.6	2.4 ± 0.6	0.1^†^	4.0
Concentric 120°/s	ACL patients	2.1 ± 0.5	1.9 ± 0.5	0.2^†^	9.5
	Active controls	2.1 ± 0.5	2.2 ± 0.4	−0.1	−4.8
	Less-active controls	2.0 ± 0.5	1.9 ± 0.5	0.1	5.0
Concentric 180°/s	ACL patients	1.9 ± 0.5	1.7 ± 0.4	0.2^†^	10.5
	Active controls	1.8 ± 0.5	1.9 ± 0.4	−0.1	−5.6
	Less-active controls	1.9 ± 0.5	1.7 ± 0.4	0.2^†^	10.5
Hamstring (Nm/kg)					
Eccentric 60°/s	ACL patients	2.4 ± 0.5	2.0 ± 0.5	0.4^†^	16.7
	Active controls	2.4 ± 0.4	2.4 ± 0.5	0.0	0.0
	Less-active controls	2.5 ± 0.6	2.3 ± 0.6	0.2	8.0
Isometric	ACL patients	1.5 ± 0.3	1.4 ± 0.4	0.1^†^	6.7
	Active controls	1.6 ± 0.4	1.6 ± 0.4	0.0	0.0
	Less-active controls	1.5 ± 0.3	1.5 ± 0.4	0.0	0.0
Concentric 60°/s	ACL patients	1.3 ± 0.3	1.2 ± 0.3	0.1	7.7
	Active controls	1.4 ± 0.4	1.4 ± 0.3	0.0	0.0
	Less-active controls	1.2 ± 0.3	1.2 ± 0.4	0.0	0.0
Concentric 120°/s	ACL patients	1.1 ± 0.3	1.1 ± 0.3	0.0	0.0
	Active controls	1.3 ± 0.4	1.2 ± 0.3	0.1	7.7
	Less-active controls	1.1 ± 0.2	1.1 ± 0.3	0.0	0.0
Concentric 180°/s	ACL patients	1.1 ± 0.3	1.1 ± 0.2	0.0	0.0
	Active controls	1.2 ± 0.3	1.2 ± 0.4	0.0	0.0
	Less-active controls	1.0 ± 0.3	1.0 ± 0.3	0.0	0.0

^†^Between-leg difference within each group (*P* < 0.05)

The MANOVA for hamstring MVCs showed a leg main effect (*F*_5,65_ = 3.3, *P* = 0.010) and a group by leg interaction (*F*_10,132_ = 2.5, *P* = 0.010). Follow-up by univariate ANOVAs showed an interaction effect for eccentric and isometric contractions (*P* ≤ 0.033) caused by the greater between-leg difference in ACL patients versus controls (Table 2).

Table 3 shows the voluntary quadriceps activation data. The MANOVA for quadriceps activation revealed a between-group difference (*F*_10,132_ = 2.1, *P* = 0.028), a leg main effect (*F*_5,65_ = 3.3, *P* = 0.011), and a group by leg interaction (*F*_10,132_ = 3.1, *P* = 0.001). CAR in ACL patients was lower than in active controls (*P* = 0.002), and there was a greater between-leg difference in ACL patients versus controls for isometric MVCs and estimated maximal force.

**Table 3:** Single-joint neuromuscular data of both legs of ACL-deficient patients and active and less-active controls (mean ± SD)

Variables	Group	Non-injured leg/dominant leg	Injured leg/non-dominant leg	Difference	
				Absolute	Percentage
Quadriceps voluntary force and muscle activation					
CAR (%)*	ACL patients	96.6 ± 2.6	95.7 ± 3.2	0.9	0.9
	Active controls	98.2 ± 1.7	98.4 ± 1.4	−0.2	−0.2
	Less-active controls	96.8 ± 2.0	97.1 ± 2.0	−0.3	−0.3
Isometric MVC (Nm)	ACL patients	206.6 ± 70.3	183.6 ± 74.3	23.0^†^	11.1
	Active controls	191.3 ± 62.3	204.7 ± 73.7	−13.4^†^	−7.0
	Less-active controls	190.2 ± 66.2	190.8 ± 71.6	−0.6	−0.3
Estimated MVC (Nm)	ACL patients	160.8 ± 54.0	142.6 ± 55.2	18.2^†^	11.3
	Active controls	144.9 ± 48.5	153.6 ± 53.3	−8.7^†^	−6.0
	Less-active controls	141.9 ± 48.1	148.3 ± 54.2	−6.4	−4.5
Potentiated doublet force (Nm)	ACL patients	81.6 ± 26.1	72.7 ± 25.6	8.9	10.9
	Active controls	74.8 ± 21.5	73.1 ± 22.1	1.7	2.3
	Less-active controls	81.7 ± 26.7	73.7 ± 24.3	8.0	9.8
Activation (% of potentiated twitch)	ACL patients	−24.3 ± 12.3	−24.7 ± 11.7	−0.4	1.6
	Active controls	−28.6 ± 9.3	−29.5 ± 7.0	−0.9	3.1
	Less-active controls	−28.8 ± 7.6	−27.5 ± 8.2	1.3	−4.5
Force accuracy (Nm)^a^					
Eccentric	ACL patients	12.1 ± 5.7	12.7 ± 5.3	−0.6	−5.0
	Active controls	9.7 ± 4.3	10.1 ± 3.9	−0.4	−4.1
	Less-active controls	12.3 ± 5.7	12.0 ± 5.8	0.3	2.4
Isometric	ACL patients	2.4 ± 2.1	2.8 ± 4.5	−0.4	−16.7
	Active controls	2.0 ± 1.9	2.0 ± 1.3	0.0	0.0
	Less-active controls	2.3 ± 2.0	2.4 ± 2.2	−0.1	−4.3
Concentric	ACL patients	10.9 ± 6.7	9.5 ± 6.9	1.4	12.8
	Active controls	7.6 ± 5.1	7.3 ± 3.2	0.3	3.9
	Less-active controls	9.2 ± 5.6	9.6 ± 6.8	−0.4	−4.3
Force variability (% of mean force)^b^					
Eccentric	ACL patients	21.0 ± 11.0	26.6 ± 16.7	−5.6	−26.7
	Active controls	20.0 ± 10.3	20.7 ± 7.5	−0.7	−3.5
	Less-active controls	24.0 ± 10.1	24.3 ± 11.1	−0.3	−1.3
Isometric	ACL patients	3.4 ± 2.6	4.6 ± 7.2	−1.2	−35.3
	Active controls	2.7 ± 1.1	3.0 ± 1.2	−0.3	−11.1
	Less-active controls	4.0 ± 2.6	3.8 ± 2.4	0.2	5.0
Concentric	ACL patients	18.8 ± 8.9	18.8 ± 9.0	0.0	0.0
	Active controls	15.7 ± 11.3	16.5 ± 7.0	−0.8	−5.1
	Less-active controls	15.6 ± 7.5	17.3 ± 10.1	−1.7	−10.9
Proprioception (°)^c^					
15°	ACL patients	3 ± 2	3 ± 3	0	0
	Active controls	4 ± 3	5 ± 3	−1	25.0
	Less-active controls	4 ± 3	6 ± 5	−2	−50.0
30°	ACL patients	4 ± 3	3 ± 3	1	25.0
	Active controls	4 ± 3	3 ± 2	1	25.0
	Less-active controls	4 ± 3	3 ± 2	1	25.0
45°	ACL patients	3 ± 3	4 ± 3	−1	−33.3
	Active controls	3 ± 3	4 ± 2	−1	−33.3
	Less-active controls	4 ± 3	4 ± 3	0	0.0
60°	ACL patients	3 ± 2	3 ± 2	0	0.0
	Active controls	4 ± 3	4 ± 3	0	0.0
	Less-active controls	4 ± 2	3 ± 2	1	25.0

*CAR* central activation ratio

* Between-group difference (*P* < 0.05)

^†^Between-leg difference within each group (*P* < 0.05)

^a^Force accuracy is expressed as the absolute difference between the produced force and the target force

^b^Force variability was quantified by the SD of the produced force divided by the mean force (i.e. coefficient of variation)

^c^Proprioception is expressed as the absolute error relative to the target position

MANOVAs did not show any statistical effects in quadriceps force accuracy and variability and knee joint proprioception (all n.s., Table 3).

### Multi-joint neuromuscular function

Table 4 shows the multi-joint neuromuscular data. The MANOVA showed no effects for static balance (all n.s.). The ANOVA for dynamic balance revealed a group effect (*F*_2,69_ = 9.0, *P* < 0.001) and group by leg interaction (*F*_2,69_ = 6.0, *P* = 0.004). Dynamic balance in ACL patients was poorer compared with controls (*P* ≤ 0.004) and showed a greater between-leg difference in ACL patients and less-active controls than active controls. The ANOVA for single-leg hop distance showed a group by leg interaction (*F*_2,69_ = 11.4, *P* < 0.001); between-leg differences were greater for ACL patients and less-active controls than active controls.

**Table 4:** Multi-joint neuromuscular data of both legs of ACL-deficient patients and active and less-active controls (mean ± SD)

Variables	Group	Non-injured leg/dominant leg	Injured leg/non-dominant leg	Difference	
				Absolute	Percentage
One-leg standing balance test, eyes open (s)	ACL patients	60 ± 0	60 ± 0	0.0	0.0
	Active controls	60 ± 0	60 ± 0	0.0	0.0
	Less-active controls	58 ± 6	57 ± 13	1.0	1.7
One-leg standing balance test, eyes closed (s)	ACL patients	33 ± 22	29 ± 20	4.0	12.1
	Active controls	31 ± 20	37 ± 20	−6.0	−19.4
	Less-active controls	26 ± 17	27 ± 20	−1.0	−3.8
Star excursion balance test, composite score (% leg length)^a,^*	ACL patients	83 ± 7	81 ± 7	2^†^	2.4
	Active controls	91 ± 13	91 ± 12	0	0.0
	Less-active controls	91 ± 10	93 ± 11	−2^†^	−2.2
Single-leg HOP test (cm)	ACL patients	139 ± 28	116 ± 34	23^†^	16.5
	Active controls	137 ± 34	134 ± 36	3	2.2
	Less-active controls	128 ± 43	121 ± 42	7^†^	5.5

* Between-group difference (*P* < 0.05)

^†^Between-leg difference within each group (*P* < 0.05)

^a^The composite score is expressed as the mean reaching distance, relative to leg length, of the eight directions

### Overall index of neuromuscular leg function

**Fig. 2:**
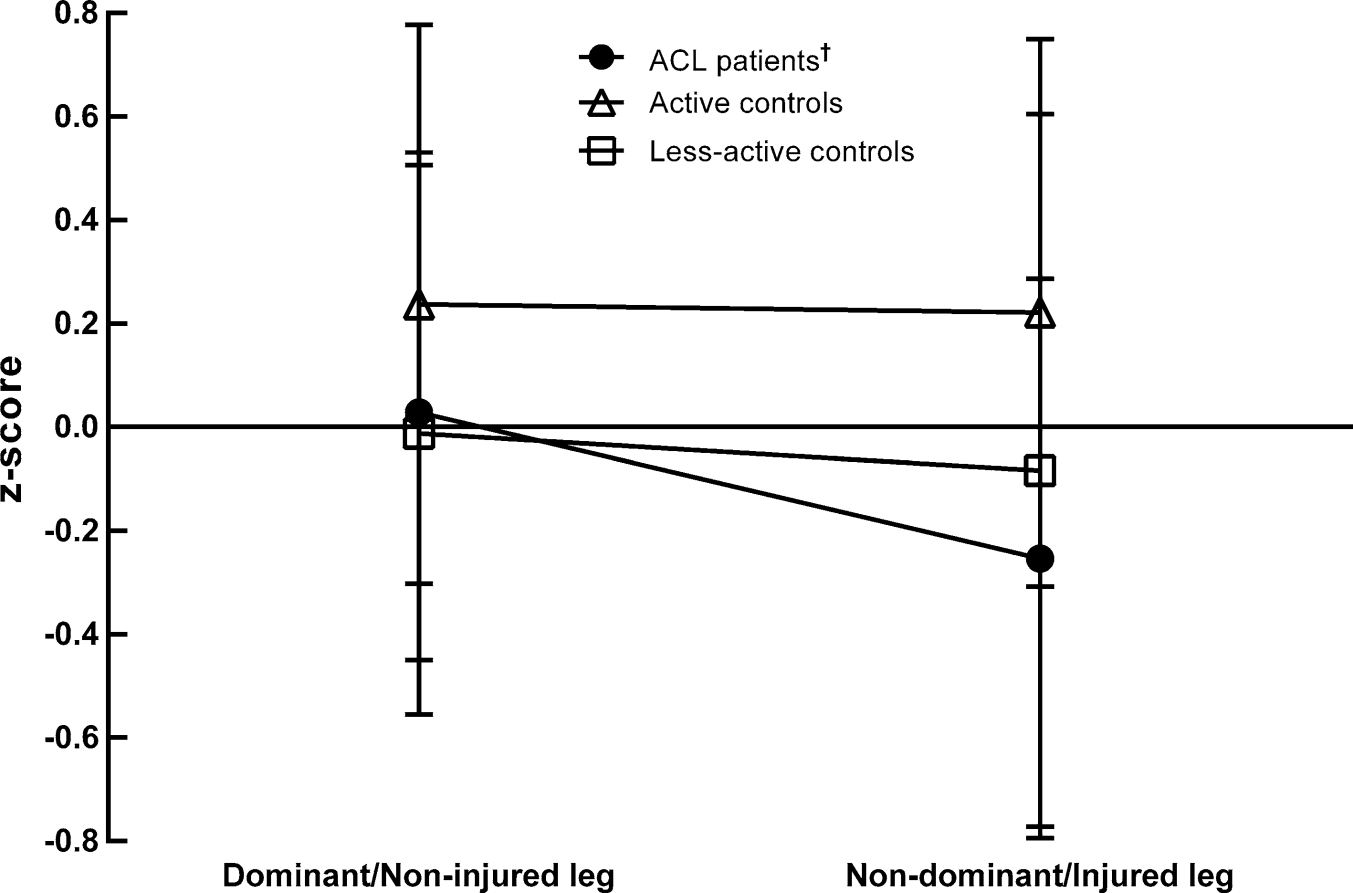
Overall index of neuromuscular function expressed as the mean z-score calculated over all neuromuscular measures. A z-score of zero reflects the mean neuromuscular function pooled across all six legs. ^†^Between-leg difference within each group (*P* < 0.05). *Note* no bilateral impairments were observed

Figure 2 illustrates the group by leg interaction effect for overall neuromuscular function (*F*_2,69_ = 7.0, *P* = 0.002) caused by better overall neuromuscular function in the non-injured leg (*P* < 0.05).

## Discussion

Several previous studies have questioned the validity of using the non-injured leg as a reference for the deficit in neuromuscular function of the injured leg [37, 43, 44]. Our data suggest that the use of the non-injured leg as reference for the injured leg’s neuromuscular function is valid except for dynamic balance tests and voluntary quadriceps activation.

## Single-joint neuromuscular function

No bilateral impairments in quadriceps strength were observed despite the reduction in physical activity after the ACL injury. The absence of bilateral weakness was unexpected because 40 days of detraining can reduce healthy subjects’ quadriceps strength by 0.3 % day^−1^ [33] and in ACL patients, bilateral quadriceps strength impairments are still apparent up to 37 days after injury [31]. We suspect the timing of the assessments after the injury is an important factor to detect bilateral quadriceps weakness because we tested ACL patients 208 days post-injury and only found strength impairments in the injured leg.

Activation failure is often cited as a mechanism underlying quadriceps weakness [26, 35] and is observed in ACL patients for as long as 119 days after injury [44]. During the rehabilitation phase, activation deficits decrease over time [39], so it is likely that the injured legs’ quadriceps weakness is caused by impaired muscle activation. In contrast to this, our twitch interpolation data did not provide evidence for an impaired voluntary drive to the quadriceps muscles after 208 days. In addition, the size of the potentiated twitch force did not indicate quadriceps muscle weakness. In addition, the CAR in ACL patients showed a 5 % activation deficit (Table 3), which is much smaller than the 14 % deficit reported 37 days after injury [31]. Our data suggest that quadriceps muscle weakness might be caused by other factors, such as an increase in hamstring coactivation [1], albeit untested in the present study.

Hamstring strength in our ACL patients’ injured leg was 9–16 % lower compared with the non-injured leg, which is consistent with previous work [10, 40]. Hamstring strength in the non-injured leg has not been examined previously in the literature, but we found no signs of weakness (Table 2). Hamstring strength appears to be an important regulator of ACL loading during athletic manoeuvres. A 25 % reduction in hamstring strength has been reported to result in a 36 % increase in ACL loading during sidestep cutting [47]. Therefore, the monitoring of hamstring strength should be prioritized to reduce the risk of ACL rupture.

Force control was not affected in ACL patients, which is surprising because previous studies have reported poor force accuracy [36] and variability [6] in patients relative to controls. These impairments in force control were also accompanied by greater hamstring coactivation [6, 36]. Other studies also report altered quadriceps activation patterns during a force control task [49–51]. Quadriceps and hamstring electromyogram activity were not measured in the present study, but we expect that muscle activation patterns would have been similar to controls because our ACL patients showed no impairments in force control.

ACL injury did not affect proprioception in either leg. Intuitively, damage to the ACL, a ligament comprising mechanoreceptors that sense the position of the knee joint should affect proprioception [45]. However, our data agree with a recent review suggesting that proprioceptive deficits in ACL patients’ injured and non-injured leg are small and not clinically meaningful [18]. Proprioception might remain unaffected due to compensation by mechanoreceptors in and around the knee joint [25] or due to a more prominent role of motor commands when mechanoreceptors in the ACL lack function [38].

## Multi-joint neuromuscular function

SEBT scores were 10–11 % lower in the injured and non-injured leg compared with control legs, where scores on the less challenging static balance test showed no between-leg differences. The SEBT is often used to quantify deficits in dynamic balance in patients with a lower extremity injury, but few such studies included ACL patients [20]. Nonetheless, one ACL study found bilateral impairments in SEBT performance prior to surgery [23], confirming our findings. It has been proposed that bilateral performance impairments can only be detected by tests that greatly stress the knee joint [15]. The SEBT exemplifies such a test, which requires not only muscle strength but also dynamic postural control.

Our study offers new information by examining hop distance prior to surgery; however, hop performance was not impaired in ACL-deficient patients. The hop test is commonly employed following ACL reconstruction, but surprisingly few studies compared the hop distance to controls [7, 30, 32]. Two of three studies reported a bilateral reduction in hop distance [7, 30], which suggests that bilateral reductions emerge after surgery because we found no bilateral impairments prior to surgery. Nonetheless, our ACL patients jumped 23 cm (0.73 SDs) less with the injured versus the non-injured leg, which might be clinically relevant because only small between-leg differences were observed for active (3 cm, 0.09 SDs) and less-active (7 cm, 0.17 SDs) controls.

## Active versus less-active controls

Little is known about how long-term training affects maximal voluntary force and the ability to control submaximal voluntary forces; however, we found no differences in single- or multi-joint neuromuscular functions between active and less-active controls. This is surprising because maximal leg strength was higher in amateur soccer players than sedentary controls [8, 13], and this difference increased with skill level of players [8]. Our less-active controls were still involved in sports although at a lower level, and fewer hours per week. Thus, it might be that our less-active controls were not inactive enough to differ significantly in neuromuscular function from active controls. Further research is needed to provide insights into how training history might affect neuromuscular functions other than maximal leg strength.

## Limitations

Dynamic balance and voluntary quadriceps activation were affected in both legs after ACL injury, but it remains possible that these impairments were already present before the injury. To determine risk factors for ACL rupture, more studies are needed to examine the bilateral neuromuscular and biomechanical function before ACL injury [16, 21, 24] and correlate these with post-ACL injury outcomes.

ACL-deficient patients in the present study were all awaiting surgery but due to several reasons some were operated on sooner than others. Acceptance of and coping with the ACL injury takes time and could have affected our performance outcomes [41]. Although it is common that the time between injury and surgery differs between patients, a more homogeneous group might have resulted in different neuromuscular outcomes [31].

Force control, proprioception, and static balance were not impaired following the ACL injury but modifications in afferent feedback [45] and cortical sensorimotor areas [3, 4, 22, 29] could have prevented these functions from deterioration. Further studies are needed to determine whether these changes in the nervous system are really compensatory mechanisms or are just side effects of the ACL injury.

## Conclusion

Whereas previous studies found bilateral impairments in early stages after an ACL injury, we have found that neuromuscular function, except for dynamic balance and voluntary quadriceps activation, was not impaired in the non-injured leg ~208 days after the injury despite the reduction in physical activity following the injury. Therapists should continue to focus on rehabilitating the injured leg following an ACL injury and the non-injured leg can serve as adequate reference to examine the recovery of the injured leg’s neuromuscular function.
